# A Novel Preparation Method of Two Polymer Dyes with Low Cytotoxicity

**DOI:** 10.3390/ma10030219

**Published:** 2017-02-23

**Authors:** Dongjun Lv, Mingjie Zhang, Jin Cui, Weixue Li, Guohua Zhu

**Affiliations:** 1 Department of Chemistry, School of Science, Tianjin University, Tianjin 300354, China; lvdongjun@tju.edu.cn (D.L.); liweixuetju@163.com (W.L.); zhuguohua@tju.edu.cn (G.Z.); 2 National Foodstuff Inspection Center, Tianjin Product Quality Inspection Technology Research Institute, Tianjin 300384, China; tjzjchem@163.com; 3 Shandong Yu Hong New Pigment Co., Ltd., Dezhou 253000, China

**Keywords:** polymer dyes, azo edible colorant, O-carboxymethyl chitosan, low cytotoxicity

## Abstract

A new preparation method of polymer dyes was developed to improve both the grafting degree of the azo dyes onto O-carboxymethyl chitosan (OMCS) and the water solubility of prepared polymer dyes. Firstly, the coupling compound of two azo edible colorants, sunset yellow (SY) and allura red (AR), was grafted onto OMCS, and then coupled with their diazonium salt. The chemical structure of prepared polymer dyes was determined by Fourier transform-infrared spectroscopy and ^1^H-NMR, and the results showed that the two azo dyes were successfully grafted onto OMCS. The grafting degree onto OMCS and the water solubility of polymer dyes were tested, and the results showed that they were both improved as expected. The UV-vis spectra analysis results showed that the prepared polymer dyes showed similar color performance with the original azo dyes. Eventually, the cytotoxicity of prepared polymer dyes was tested and compared with the original azo dyes by a cytotoxicity test on human liver cell lines LO2, and the results showed that their grafting onto OMCS significantly reduced the cytotoxicity.

## 1. Introduction

The addition of food dyes into the processing of foodstuffs is a common strategy to improve the sensory properties, color and commercial characteristics [1,2,3]. Generally, most food dyes widely used in China and abroad are synthetic azo dyes, and they are applied in fields such as foodstuffs, drugs and cosmetics [4] due to their high brightness and wide color spectrum. However, the excessive and long-term consumption of these dyes is very harmful to human health, particularly when the excessive usage causes food safety issues [5,6,7]. As the common edible colorant, sunset yellow (SY) and allura red (AR) have strong genetic toxicity [8,9,10]. It is found that AR has potential behavioral effects on humans and animals and it will increase hyperactivity of children [11,12,13]. Similarly, SY shows negative effects on the cellular immunity [14]. Additionally, both of them will induce DNA damage when their intake amount exceeds the acceptable daily intake (ADI). It was reported that the harm of the above two edible colorants was attributed to the aromatic amine and amide in their degradation products, and the ADI of SY and AR were severely limited at 0–1 and 7 mg/kg body weight respectively [15,16,17]. With the growing concern regarding food safety issues, the improvement of the safety of edible colorants has received extensive attention. Up to now, the azo dyes are still the most common edible colorants that are reduced by dihydronicotinamide adenine dinucleotide (NADH) [18,19] in the liver but this metabolized reduction produces aromatic amines that cause carcinogenicity and gene toxicity.

Based on what is stated above, the limited usage of traditional synthetic edible colorants has become a global consensus, and in some European countries, laws and regulations on the restricted usage amount and species of edible colorants in foodstuffs have been passed. Thus, it is important to seek some new species of edible colorants with high safety, high stability and convenient application. Polymeric dyes, as functional colored polymers, possess both the color feature of organic dyes and high stability of the polymers. Additionally, due to their macromolecule structure, they have excellent properties of heat and chemical stability, biocompatibility, good light fastness [20,21] and high resistance to gastrointestinal absorbance, which improves their safety in human bodies [22]. Generally, in the preparation of polymer dyes, the carboxymethyl chitosan and its derivatives are selected as the optimal carrier because of their good water solubility [23,24], enhanced antibacterial property [25], biodegradability, improved biocompatibility [26,27], non-toxicity and safety for humans [28]. Additionally, the –NH_2_ and –COOH group in their chemical structure can provide the activated site for the conjunction with dyes.

In our previous paper, two azo dyes, SY and AR, were sulfonyl-chloridized firstly by a chlorosulfonylation reaction and then grafted to OMCS through a Schotten–Baumann reaction [29]. However, the grafting degree of the azo dyes onto OMCS was lower because of the steric hindrance between azo dyes and OMCS. Additionally, the water-soluble sulfonic acid group in the azo dyes was removed, which resulted in a decrease in the water solubility of prepared polymer dyes. Thus, in order to improve the grafting degree of the azo dyes onto OMCS and the water solubility of prepared polymer dyes, we changed the synthesis route of polymer dyes and retained the sulfonic acid group of azo dyes. In the novel synthesis route of polymer dyes, the coupling component of the azo edible colorants, SY and AR, was firstly grafted onto O-carboxymethyl chitosan (OMCS), and then coupled with their diazonium salt to prepare polymer dyes. The grafting degree of SY and AR onto OMCS and the water solubility of prepared polymer dyes were compared with those in our previous work [29]. In addition, the chemical structure of prepared polymer dyes was determined by Fourier transform infrared spectroscopy (FT-IR) and ^1^H-NMR spectra, and their toxicity was tested and compared with the original azo edible colorant by a cytotoxicity experiment.

## 2. Results and Discussion

### 2.1. Chemical Structure Characterization of Prepared Polymer Dyes

**Scheme 1:**
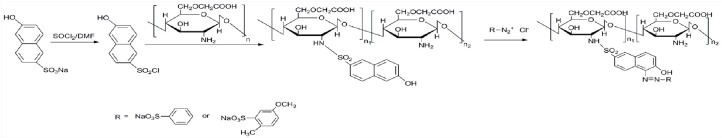
Schematic of the synthesis process of polymer dyes.

From what has been mentioned in the preparation process, the schematic synthesis process of polymer dyes was illustrated in Scheme 1. The sulfonyl group in 2-naphthol-6-sodium sulfonate—the coupling compound of the two azo dyes—was chlorinated to form a sulfonyl chloride group, which was reacted with the amino group in OMCS to form a –SO_2_NH– group. Then, the coupling compound grafted onto OMCS reacted with diazonium salt to fabricate the polymer dyes. In the synthesis process, the sulfonyl group in diazonium salt was well retained, which was beneficial for the improvement of the water solubility of prepared polymer dyes.

#### 2.1.1. FT-IR Analysis

**Figure 1:**
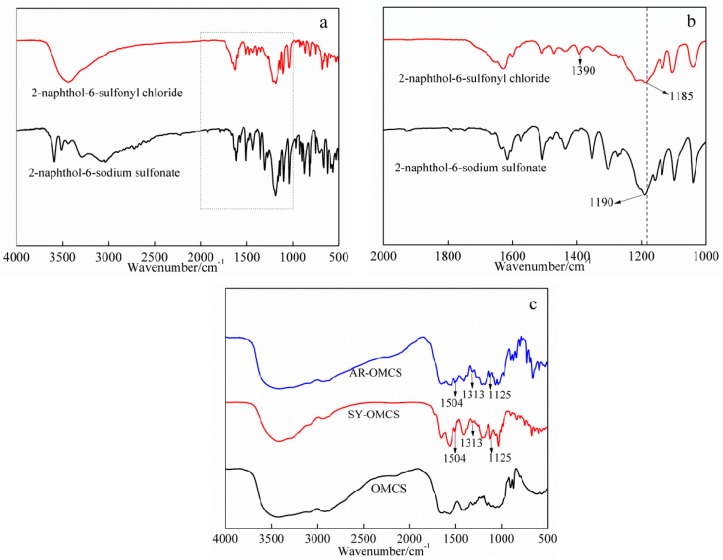
FT-IR spectra of 2-naphthol-6-sodium sulfonate and 2-naphthol-6-sulfonylchloride in the wavenumber range 500–4000 cm^−1^ (**a**) and in the wavenumber range 1000–2000 cm^−1^ (**b**); and OMCS, SY-OMCS and AR-OMCS (**c**).

The chemical structure of the coupling compound 2-naphthol-6-sodium sulfonate, the intermediate product 2-naphthol-6-sulfonylchloride, OMCS and the prepared polymer dyes were analyzed by FT-IR, and the results are shown in Figure 1.

The FT-IR spectra of 2-naphthol-6-sodium sulfonate and 2-naphthol-6-sulfonylchloride are shown in Figure 1a. In order to investigate the chemical structure of the intermediate product 2-naphthol-6-sulfonylchloride, infrared spectroscopy in the wavenumber range 1000–2000 cm^−1^ was observed on a large scale, and the results are shown in Figure 1b; in the curve of 2-naphthol-6-sulfonylchloride, two characteristic adsorption peaks at 1390 and 1185 cm^−1^ corresponded to the asymmetric and symmetric stretching vibrations of the S=O bond of the –SO_2_Cl group were observed, suggesting that the coupling compound 2-naphthol-6-sodium sulfonate was sulfonylchlorided successfully.

It could be seen from the Figure 1b that the prepared polymer dyes had a similar adsorption peak with that of OMCS, and there were some new adsorption peaks in the curve of SY-OMCS and AR-OMCS. The characteristic peak at 1504 cm^−1^ corresponded to the stretching vibration adsorption peak of –N=N– in the azo dyes [30], and the characteristic peaks at about 1313 and 1125 cm^−1^ corresponded to the S=O asymmetric and symmetric stretching vibrations adsorption peak in the –SO_2_NH– group that formed between the azo dyes and OMCS [31]. Thus, the FT-IR analysis results illustrated that the two edible colorants, SY and AR, were successfully grafted onto OMCS.

#### 2.1.2. ^1^H-NMR Analysis

**Figure 2:**
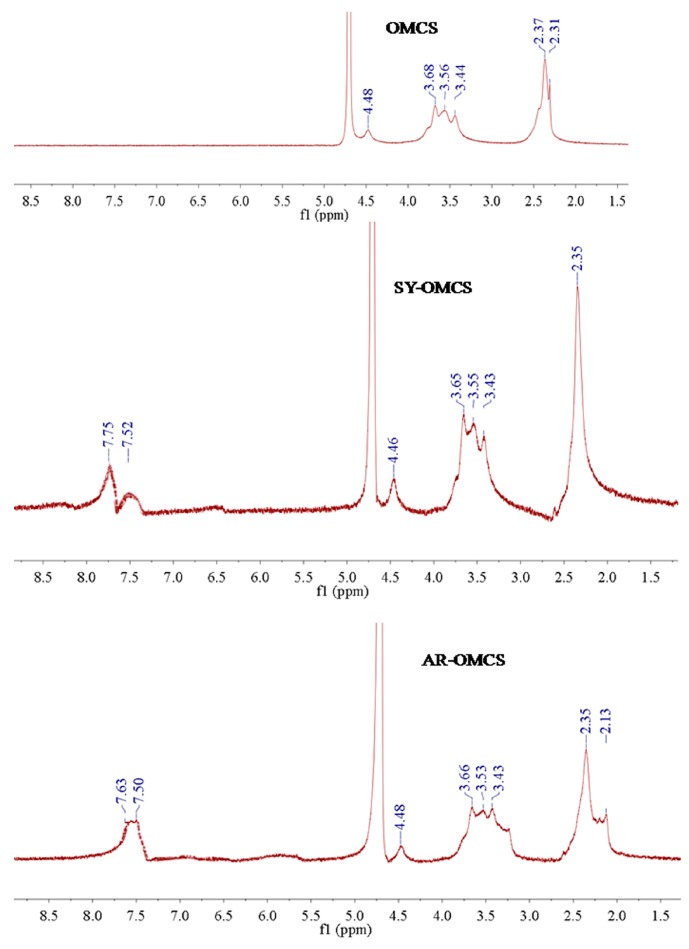
^1^H-NMR spectrum of OMCS and prepared polymer dyes.

The ^1^H-NMR spectra analysis results of prepared polymer dyes and OMCS are shown in Figure 2. It could be seen from the ^1^H-NMR spectra of OMCS that the proton assignments were shown as follows: 2.31–2.38 ppm (CH, carbon 2 of glucosamine ring), 3.43–3.68 ppm (carbon 1, 3, 4, 5 and 6 of glucosamine ring), 4.48 ppm (CH, carbon 7 of carboxymethyl groups). In the ^1^H-NMR spectra of two polymer dyes, the new characteristic proton signals in the range of 7.52–7.75 ppm and 7.50–7.63 ppm, corresponding to SY and AR respectively, were observed, which indicated that SY and AR were successfully grafted onto OMCS.

### 2.2. Grafting Degree and Water Solubility of Prepared Polymer Dyes

#### 2.2.1. Grafting Degree Determination by Element Analysis

The grafting degrees of azo dyes on OMCS were determined by elemental analysis, and the results were listed in Table 1. For the SY-OMCS, it could be calculated from the element content of N and S that the atomic ratio of N/S was 5.5:2. There were two S and N atoms in the SY molecule, and one N atom in every OMCS unit (Mw = 219), thus it was concluded that there was one SY molecule in every 3.5 OMCS units. Meanwhile, it could be worked out that in AR-OMCS the atomic ratio of N/S was 4.5:2, and there was one AR molecule in every 2.5 OMCS units. Based on the analysis results mentioned above, the grafting degree of SY and AR onto OMCS could be calculated as 35.1% and 45.7%, which was higher than that in our previous study (29.1% and 33.3% respectively) [29]. Thus, the novel preparation method of polymer dyes mentioned in this paper improved the grafting degree of SY and AR onto OMCS as expected.

#### 2.2.2. Effect of Amidation Reaction Conditions on the Grafting Degree of SY and AR onto OMCS

**Figure 3:**
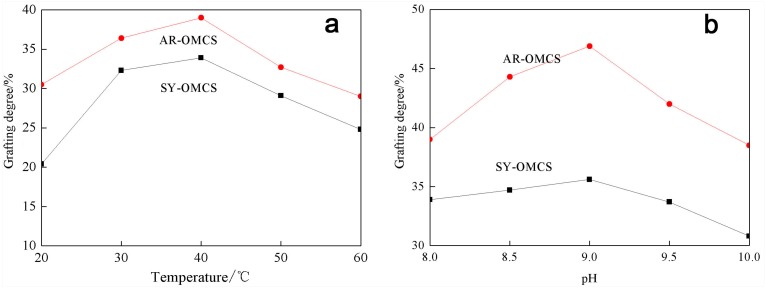
Effect of the amidation reaction conditions on the grafting degree of SY and AR onto OMCS.

Figure 3 presents the effects of amidation reaction conditions on the grafting degree of SY and AR onto OMCS. It could be seen from Figure 3a that at an amidation reaction pH of 8, the grafting degree firstly increased and then decreased when the reaction temperature increased from 20 to 60 °C. Under a reaction temperature of 40 °C, both SY and AR obtained the highest grafting degree of 33.9% and 39.0% respectively. As was well known, the increase in reaction temperature improved the reaction activity, and thus the grafting degree was consequently enhanced. Nevertheless, the hydrolysis of sulfonyl chloride intermediates was intensified at a high reaction temperature, which led to a decrease in the grafting degree of SY and AR onto OMCS.

From Figure 3b, it could be found that when the reaction temperature was fixed at 40 °C, the grafting degree of SY and AR onto OMCS firstly increased and then decreased under a ranged reaction pH between 8 and 10. When the reaction pH value was lower than 9, the nucleophilic reaction between 2-naphthol-6-sulfonylchloride and OMCS was limited, which led to a decrease in the grafting degree. In addition, the 2-naphthol-6-sulfonylchloride would be hydrolyzed with the increase in reaction pH value, as a result, the grafting degree decreased. According to what is stated above, the conclusion was made that the optimal reaction pH for the grafting of SY and AR onto OMCS was 9, and the highest grafting degree could reach up to 35.6% and 46.8% respectively.

#### 2.2.3. Water Solubility Measurement of Prepared Polymer Dyes

The water solubility of prepared polymer dyes and those prepared in our previous work [29] were tested and compared, and the results were shown in Table 2. It could be seen that in 100 g water, the solubility of SY-OMCS and AR-OMCS was 9.2 and 7.4 g respectively, which was obviously higher than those prepared in our previous work (5.7 g for SY-OMCS and 4.2 g for AR-OMCS). Thus, it was concluded that in our novel preparation method, the retention of the sulfonic group in the azo dyes improved the water solubility of prepared polymer dyes as expected.

### 2.3. UV-Vis Spectra Analysis of Prepared Polymer Dyes

**Figure 4:**
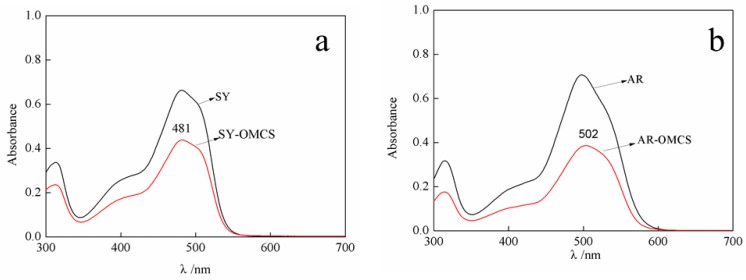
UV-vis spectra of prepared polymer dyes and the original azo dyes. (**a**) SY and SY-OMCS; (**b**) AR and AR-OMCS.

The color performance of prepared polymer dyes was determined and compared with original azo dyes by UV-vis spectra analysis, and the results were shown in Figure 4. It was obvious that the prepared polymer dyes had similar adsorption spectra with the original azo dyes, and the λ_max_ of SY-OMCS and AR-OMCS was 481 and 502 nm respectively. From what is stated above, it could be considered that the grafting onto OMCS did not change the color performance of prepared polymer dyes, and they would show similar performance to the original azo dyes in the dyeing operation.

### 2.4. Cytotoxicity Test of Prepared Polymer Dyes

**Figure 5:**
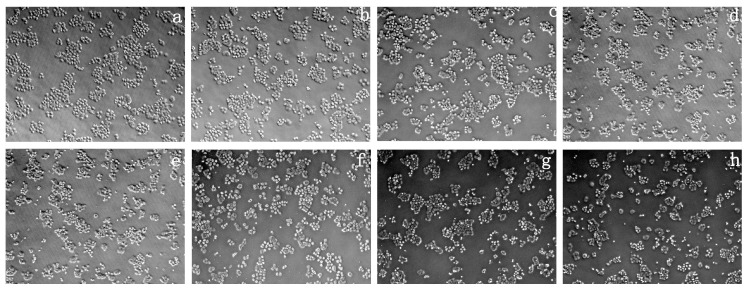
Performance of human hepatic cells (LO2) treated with various concentrations of SY and SY-OMCS: (**a**) control SY; (**b**) 0.25 g/L SY; (**c**) 0.5 g/L SY; (**d**) 1 g/L SY; (**e**) control SY-OMCS; (**f**) 1 g/L SY-OMCS; (**g**) 2 g/L SY-OMCS; (**h**) 4 g/L SY-OMCS.

**Figure 6:**
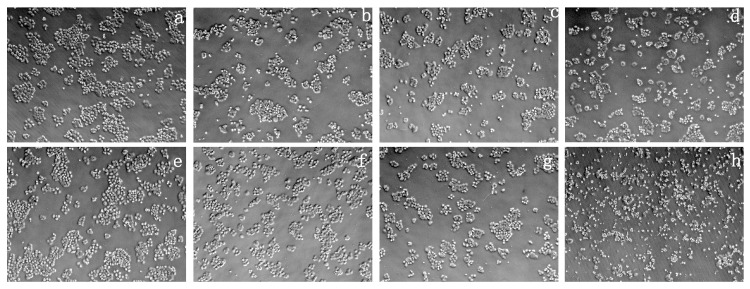
Performance of human hepatic cells (LO2) treated with various concentrations of AR and AR-OMCS: (**a**) control AR; (**b**) 0.25 g/L AR; (**c**) 0.5 g/L AR; (**d**) 1 g/L AR; (**e**) control AR-OMCS; (**f**) 1 g/L AR-OMCS; (**g**) 2 g/L AR-OMCS; (**h**) 4 g/LAR-OMCS.

The cytotoxicity of prepared polymer dyes was tested and compared with the original azo dyes by a cytotoxicity test on normal human liver cell lines LO2. From the observation results obtained by a fluorescent inverted microscope, it was found that both the original azo dyes and prepared polymer dyes induced vague cellular morphology and cell shrinkage in a concentration-depended manner (Figure 5 and Figure 6). Furthermore, it was obvious that in order to achieve the same cell inhibition effect, the concentration of prepared polymer needed to be higher than that for the original azo dyes.

**Figure 7:**
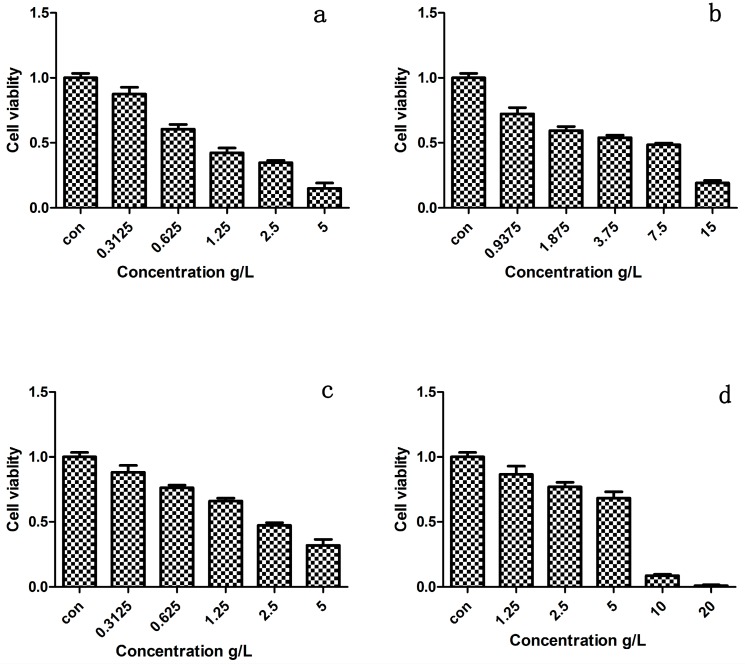
Cell viability of normal cells (LO2) treated with SY (**a**); SY-OMCS (**b**); AR (**c**) and AR-OMCS (**d**).

Additionally, a MTT (3-[4,5-dimethylthiazol-2-yl]-2,5-diphenyltetrazolium bromide) assay was done and the results were shown in Figure 7. It could be found that the LO2 cell viability showed a dependence on the dye concentration, and the higher the dye concentration was, the lower the cell viability was. Additionally, the prepared polymer dyes showed an obvious lower cytotoxicity than the original azo dyes, which was due to the fact that OMCS was safe on the normal human liver cell lines LO2 [32] and due to the impediment in the absorbance of the azo dyes by the cells’ membranes. When the SY concentration reached up to 1.25 g/L, the cell viability was below 0.5, however, for SY-OMCS, the cell viability was still up to 0.5 even when the SY-OMCS concentration reached up to 3.75 g/L. A similar trend for AR and AR-OMCS was observed. The cell viability was 0.48 when the AR concentration was 2.5 g/L, and when the AR-OMCS concentration was 5 g/L, the cell viability was still up to 0.77. All the results mentioned above illustrated that the grafting onto OMCS significantly reduced the cytotoxicity of the azo dyes. Based on the MTT assay results, the concentration required for 50% inhibition of cell viability IC50 was calculated with Graphpad prism 5 software (V5.01, GraphPad Software, Inc., La Jolla, CA, USA), and the results are shown in Table 3. The IC50 values of SY, SY-OMCS, AR and AR-OMCS on LO2 were 1.105, 3.981, 2.298 and 5.513 g/L respectively. It was obvious that the IC50 values for prepared polymer dyes were 2.4–3.6 times higher than that for the original azo dyes edible, illustrating that the grafting onto OMCS dramatically reduced the cytotoxicity of the azo dyes on the liver cell lines LO2.

## 3. Materials and Methods

### 3.1. Material

The 2-naphthol-6-sodium sulfonate (90% purity) and p-aminobenzene sulfonic acid (98% purity) were purchased from Shandong XiYa Chemical industry Co., Ltd. (Linshu, China), and O-Carboxymethyl chitosan (OMCS, Mw = 500,000 g/mol) was purchased from Shanghai Dibo Chemical Technology Co., Ltd. (Shanghai, China) In addition, the 4-amino-5-methoxy-2-methylbenzesulfonic acid (90% purity) was provided by J&K Scientific Co., Ltd. (Beijing, China) The other reagents were analytical grade, provided by Shandong XiYa Chemical industry Co., Ltd. and used without purification.

### 3.2. Methods

#### 3.2.1. Preparation of Diazonium Salt

Under magnetic stirring, the 4-aminobenzenesulfonic acid (0.65 g, 3.7 mmol) was added into 20 mL water and dissolved at 40 °C. Then, the HCl (31% w/w, 1.4 g, 11.9 mmol) and NaNO_2_ (0.27 g, 3.76 mmol) aqueous solution with a concentration of 30% (w/w) was added into the aforementioned solution. After the reaction was completed, the excessive NaNO_2_ was removed by adding sulfamic acid to obtain the diazonium salt solution. Similarly, the diazonium salt solution of 4-amino-5-methoxy-2-methylbenzensulfonic acid was obtained, and the dosages of 4-amino-5-methoxy-2-methylbenzensulfonic acid, NaNO_2_ and HCl (31% w/w) were 0.9 g (3.7 mmol), 0.27 g (3.76 mmol) and 1.4 g (11.9 mmol) respectively.

#### 3.2.2. Preparation of Sulfonyl Chloride

The 2-naphthol-6-sodium sulfonate was purified as the procedure: 2-naphthol-6-sodium sulfonate (20 g) was dissolved in 200 mL distilled water, concentrated by a rotary evaporator, filtered, washed with anhydrous ethanol and then dried at 60 °C. The purified 2-naphthol-6-sodium sulfonate (4.92 g, 20 mmol) was dispersed in a mixture of 50 mL dichloroethane and 2 mL *N*,*N*-dimethylformamide (DMF), and then 5 mL thionylchloride was dropwise added into the solution. After magnetic stirring for 30 min at room temperature, the solution was heated and kept at 60 °C for 4 h. When the reaction was completed, the thionylchloride was recycled by vacuum distillation, and the residue was cooled down and poured into ice water. Then, the mixture was filtered, washed with ice water and dichloroethane, and the filter cake was dried to obtain 2-naphthol-6-sulfonylchloride.

The chemical structure of obtained products was confirmed by Fourier translation infrared spectrum (FT-IR, Nicolet i5, Waltham, MA, USA) and ^1^H-NMR spectra (AM-400, Bruker, Karlsruhe, Germany), and the ^1^H-NMR data of 2-naphthol-6-sulfonyl chloride were shown as follows: ^1^H-NMR (400 MHz, DMSO) δ 8.02 (s, 1H), 7.80 (d, J = 8.6 Hz, 1H), 7.61 (s, 2H), 7.14–7.08 (m, 2H).

#### 3.2.3. Preparation of Polymer Dyes

Firstly, under magnetic stirring, 1.31 g OMCS was dissolved in the mixture of 25 mL H_2_O and 0.06 g nekal BX in a three-neck round bottle, and the solution pH was adjusted to 8.5–9.0 with sodium carbonate solution (10%, w/w). Then, the sulfonyl chloride product (0.97 g, 4 mmol) prepared earlier was dispersed in 10 mL tetrahydrofuran and then dropwise added into the OMCS solution. The whole process was proceeded by vigorous stirring; the temperature of 40 °C and the pH were kept stable by sodium carbonate solution, and the whole process was completed within 1 h. After reaction for another 3 h, the coupling solution was prepared.

Eventually, the polymer dyes were prepared through a coupling reaction by dropwise adding the diazonium salt solution into the coupling solution within 40 min, and the reaction pH was kept at 9.0 with the sodium carbonate solution. After completion of the coupling reaction and another 120 min stirring, the reaction solution was heated to 60 °C, kept for 30 min and then turned into a round bottle for post-processing. The water was removed by vacuum distillation to obtain a concentrated solution of polymer dyes, which was then put into a benzoylated cellulose membrane (MWCO 3500 g/mol) and placed in water to remove the impurities with low molecular-weight.

#### 3.2.4. Determination of Grafting Degree of the Azo Dyes onto OMCS

The grafting degree of the azo dyes onto OMCS was determined according to the method described in our previous work [29] and analyzed by the Elemental (C, H, N, S) analysis on a Vario EL III Elemental analyzer (Elementar, Hanau, Germany).

#### 3.2.5. Water Solubility Measurement of Prepared Polymer Dyes

The water solubility of prepared polymer dyes was measured using the procedure shown as follows: Firstly, the prepared polymer dyes’ aqueous solution with a concentration of 2.5 g/L was prepared and was diluted by n_1_ times; then, its absorbance A_1_ at the maximum absorption wavelength of polymer dyes was tested by a UV-Vis spectrophotometer. Secondly, the saturated polymer dyes’ aqueous solution was fabricated and diluted by n_2_ times, and its absorbance A_2_ at the maximum absorption wavelength of polymer dyes was tested. Then, the solubility of the prepared polymer dyes in 100 g water was calculated by the following Equation (1).

Solubility/g = A_2_ × n_2_ × 2.5/(A_1_ × n_1_ × 10) g (1)

#### 3.2.6. Characterization of Prepared Polymer Dyes

The prepared polymer dyes were characterized by Fourier translation infrared spectrum (FT-IR, Nicolet i5, America) and a ^1^H-NMR Spectrometer (AM-400, Bruker, Germany). The UV-vis spectra of the prepared polymer dyes and the original azo edible colorants were tested by a UV-Vis spectrophotometer (T9, Beijing Purkinje General Instrument Co., Ltd., Beijing, China).

#### 3.2.7. Cytotoxicity in LO2 Cell Line

The in vitro cytotoxicity investigation was carried out by MTT assays. For cytotoxicity experiments, confluent human normal liver cells (LO2) in good condition were cultured in 96-well plates (5 × 10^3^ cells/well) and dealt with various concentrations of regents at 37 °C for 24 h. Then, the cells were incubated with 20 μL MTT reagent with a concentration of 5 mg/mL for 4 h at 37 °C, dissolved in 150 μL dimethyl sulfoxide and shaken for 5 min. Finally, the light absorption (OD) of the dissolved cells was measured at 490 nm. The concentration of the two edible colorants and prepared polymer dyes required for 50% inhibition of cell viability IC50 was calculated with the Graphpad prism 5 software.

## 4. Conclusions

Based on our previous work, we developed a novel preparation method of polymer dyes to improve both the grafting degree of the azo dyes onto OMCS and the water solubility of prepared polymer dyes. In this work, the same two polymer dyes were prepared with two steps. Firstly, the coupling compound of the azo dyes, SY and AR, was grafted onto OMCS through an acyl chloride reaction, and then coupled with their diazonium salt to prepare polymer dyes. The analysis results of FT-IR, ^1^H-NMR and element analysis showed that the SY and AR were successfully grafted onto OMCS, and their grafting degree onto OMCS reached up to 35.6% and 46.8% respectively. Additionally, in 100 g water, the solubility of SY-OMCS and AR-OMCS was 9.2 and 7.4 g respectively. Both the grafting degree of the azo dyes onto OMCS and the water solubility of prepared polymer dyes were improved as expected. The UV-vis spectra analysis results showed that the prepared polymer dyes had a similar adsorption property with the original azo dyes, suggesting that prepared polymer dyes possessed similar color performance to original dyes. As for the application properties of prepared polymer dyes in the dyeing operation, this will be studied in our future work.

The vitro cell cytotoxicity test results showed that the IC50 values on human liver cell lines LO2 of prepared polymer dyes, SY-OMCS and AR-OMCS (3.981 and 5.513 g/L respectively), were obviously higher than that for the original azo dyes, SY and AR (1.105 and 2.298 g/L respectively), suggesting that the grafting onto OMCS dramatically reduced the cytotoxicity of the azo dyes.

## Figures and Tables

**Table 1:** Elemental analysis results of prepared polymer dyes.

Sample	Elemental Content/%			
	C	N	S	H
OMCS	41.2	6.5	-	5.3
SY-OMCS	38.0	4.9	4.1	4.8
AR-OMCS	36.9	5.0	5.1	5.1

**Table 2:** Water solubility of polymer dyes.

Sample	SY-OMCS	SY-OMCS ^1^	AR-OMCS	AR-OMCS ^1^
Solubility	9.2	5.7	7.4	4.2

^1^ Polymer dyes prepared in our previous work [29].

**Table 3:** IC50 values of edible colorants on human liver cell lines (LO2).

Compounds	SY	SY-OMCS	AR	AR-OMCS
IC50 (g/L)	1.105	3.981	2.298	5.513
